# Before it is too late: professional responsibilities in late-onset Alzheimer’s research and pre-symptomatic prediction

**DOI:** 10.3389/fnhum.2014.00921

**Published:** 2014-11-20

**Authors:** Silke Schicktanz, Mark Schweda, Jesse F. Ballenger, Patrick J. Fox, Jodi Halpern, Joel H. Kramer, Guy Micco, Stephen G. Post, Charis Thompson, Robert T. Knight, William J. Jagust

**Affiliations:** ^1^Department of Medical Ethics and History of Medicine, University Medical Center GöttingenGöttingen, Germany; ^2^Lichtenberg Kolleg, University of GöttingenGöttingen, Germany; ^3^Health Administration Department, Drexel UniversityPhiladelphia, PA, USA; ^4^Institute for Health & Aging, University of CaliforniaSan Francisco, San Francisco, CA, USA; ^5^UC Berkeley-UCSF Joint Medical Program, School of Public Health, University of CaliforniaBerkeley, Berkeley, CA, USA; ^6^Memory and Aging Center, Sandler Neurosciences Center, University of CaliforniaSan Francisco, San Francisco, CA, USA; ^7^Compassionate Care & Bioethics, Stony Brook MedicineStony Brook, NY, USA; ^8^Department of Gender and Women’s Studies, University of CaliforniaBerkeley, Berkeley, CA, USA; ^9^Department of Sociology, London School of EconomicsLondon, UK; ^10^Department of Psychology, University of California, BerkeleyBerkeley, CA, USA; ^11^Helen Wills Neuroscience Institute, University of CaliforniaBerkeley, Berkeley, CA, USA

**Keywords:** late-onset Alzheimer’s dementia, medical ethics, recommendations, biomarker, public health policy, research participation, cultural diversity, public engagement

## Abstract

The development of a wide array of molecular and neuroscientific biomarkers can provide the possibility to visualize the course of Alzheimer’s disease (AD) at early stages. Many of these biomarkers are aimed at detecting not only a preclinical, but also a pre-symptomatic state. They are supposed to facilitate clinical trials aiming at treatments that attack the disease at its earliest stage or even prevent it. The increasing number of such biomarkers currently tested and now partly proposed for clinical implementation calls for critical reflection on their aims, social benefits, and risks. This position paper summarizes major challenges and responsibilities. Its focus is on the ethical and social problems involved in the organization and application of dementia research, as well as in healthcare provision from a cross-national point of view. The paper is based on a discussion of leading dementia experts from neuroscience, neurology, social sciences, and bioethics in the United States and Europe. It thus reflects a notable consensus across various disciplines and national backgrounds. We intend to initiate a debate on the need for actions within the researchers’ national and international communities.

## Introduction

Alzheimer’s disease (AD) is a substantial individual threat and a major public-health challenge (WHO, 2012). Until now, there is no preventive measure or curative therapy for late-onset AD (LOAD), and symptom relief is limited (Daviglus et al., 2010). New research approaches aim at delaying onset of LOAD through treatment of “at-risk individuals”. This mirrors a general shift from pathological diagnosis to assessment of risk factors: Prediction and the start of treatment are now considered plausible approaches at the pre-symptomatic phase (Sperling et al., 2011, 2013; Shim and Morris, 2011).

Biomarkers for the identification of at-risk healthy individuals include genetic testing of APOE *ɛ*4 (Keage et al., 2010) as well as vascular risk factors (Kivipelto et al., 2005). Neurological biomarkers comprise positron emission tomography (PET) measures of beta-amyloid plaques (Aβ) (Nordberg et al., 2013), cerebrospinal fluid (CSF levels of Aβ and of total-and phosphorylated-tau) (Visser et al., 2009; Landau and Frosch, 2014), functional and structural magnetic resonance imaging (MRI), e.g., hippocampal volume (Filippi et al., 2012), and biomarkers reflecting metabolic or inflammatory changes associated with LOAD (Mapstone et al., 2014). Several of these biomarkers might predict LOAD even at the mild cognitive impairment (MCI) (Petrella et al., 2011; Prestia et al., 2013) or pre-MCI stage (Parra et al., 2010). Since some examinations are invasive, time-consuming, expensive, or ethically problematic, neuro-psychological and cognitive tests (e.g., MiniMental-State (MMSE)) (Jessen et al., 2011; Palmqvist et al., 2012) or blood tests (Mapstone et al., 2014) are also explored as low-cost-low-risk tests for potential public screening. The likely dynamic of different biomarkers during the pathophysiological process of LOAD is a major challenge (Jack et al., 2013) and still requires extensive validation of the various markers (Albert et al., 2011).

In summary, the stages discussed include a long asymptomatic stage where healthy or minimally symptomatic individuals have different (genetic, physiological, or molecular) risk factors, followed by a pre-dementia stage with mild symptoms that can be differentiated into subjective cognitive impairment (SCI) or MCI, and, finally, a symptomatic clinical dementia stage. For each of these different stages, biomarkers like the ones mentioned above are being explored and need to be validated in view of their use for dementia prevention. Three major levels are distinguished that parallel the three stages of disease development (Wright et al., 2009; Kryscio, 2014): *Primary prevention* addresses the healthy public; *secondary prevention* aims at early detection of disease by screening biomarkers to apply treatment that can stop progression. *Tertiary prevention* follows the clinical diagnosis with the aim of slowing down progression or reducing complications. The biomarkers as well as the diagnosis of the prodromal stage need to be validated, reproduced, standardized, and tested for predictive value in longitudinal studies. However, the potential for biomarkers to identify individuals who are asymptomatic and could benefit from therapies (not yet available) has revolutionized thinking about the approach to dementia care and changed the landscape of clinical trials for dementia.

This trend towards pre-symptomatic prediction has recently evoked strong ethical and sociological criticism (Le Couteur et al., 2013; Lock, 2013). Thus, David Le Couteur and his colleagues argue that it will mainly lead to overdiagnosis of AD and will therefore rather harm people. It is important to note that the development of biomarkers for LOAD occurs in an environment that, so far, largely lacks systematic ethical reflection. There is not even a specific ethical framework that could help politicians, public health decision makers, healthcare professionals, and families to deal with people at risk for developing dementia. Since biomarker research for dementia is still in its infancy, this is a critical point in time for developing an appropriate ethical framework in view of current research and future clinical implementation. The potential of current biomarker research to challenge understandings and clinical practices regarding LOAD should not be underestimated (Waldemar et al., 2007). In addition to the above mentioned future aim of primary, secondary, and tertiary prevention, some biomarkers have been approved by regulatory agencies and are, at least theoretically, available for clinical application. Such biomarkers could theoretically be used for predictive and screening purposes. This could occur if patients request these tests or if these tests were publicly promoted, e.g., as part of a so-called national dementia strategy.

Important questions that have not found sufficient consideration, so far, are related to the value and risk of knowing for different actors such as potentially affected persons, their families, the medical profession, and the various national healthcare systems (Karlawish, 2011). Furthermore, the current scholarly debate has to expand its scope beyond the setting of Western, industrialized countries. While healthcare policies and legal frameworks are contextualized in national settings, the research community is increasingly globalized. The resulting tensions have not received sufficient consideration, so far. They pose new problems and emphasize our responsibility as professionals and scientists to act as mediators between local contexts and global developments.

The following interdisciplinary considerations identify four different ethical and social dimensions, thus systematizing the current debate around central problems, controversies, and open questions in order to promote future research initiatives and open a more cross-cultural perspective: 1. healthcare and research policy balancing between research for early diagnosis and care provision for existing patients, 2. ethical issues related to participation in dementia research and disclosure of information on pre-dementia stages such as MCI, 3. long-term implications of dealing with predictive information on a personal and social level in different cultures and healthcare systems, and 4. enhancing the dialog between scientists, the public, and persons being directly affected.

## Public policy goals and concerns

### Rationale

Late-onset AD has become the target of many national action plans worldwide, e.g., in Australia, France, the Netherlands, Norway, and England. The challenge of the research lies in the complexity of progressive, multi-dimensional dementing syndromes: First, the aging brain can show multiple forms of pathological change causing dementia progression. Second, even in LOAD, different neuropathological factors, such as amyloid plaques, neurofibrillary tangles, and synaptic alterations, have been identified, but the contribution of each to the clinical picture is controversial. Third, LOAD often shows mixed etiology with vascular diseases. Finally, relationships between the genetic and neuronal level on the one hand and the cognitive and behavioral level on the other have not been established.

A variety of approaches are employed to diagnose LOAD (Waldemar et al., 2007). Genetic ApoE-tests are not recommended due to their low predictive value (Goldman et al., 2011). The major paradigm shift is to identify brain changes as biomarkers for predicting cognitive decline at preclinical stages (Albert et al., 2011; Hampel et al., 2014). Even among AD researchers, the concept of pre-symptomatic AD is being debated and requires validation. Although methods of neuroimaging to detect disease specific pathological brain changes might offer better prediction than genetic testing, they also raise important public health concerns regarding resource allocation.

### Recommendations

In the framework of democratic regulation of research and healthcare, shifts in agenda and priority setting require public information and debate. If early diagnosis is to be clinically employed, there is a need to validate the biomarkers and to identify possible co-factors explaining variation between pathological and symptomatic levels. However, an exclusive focus of public policy in dementia research on early detection raises concerns. First, it is not clear whether efficient treatment with acceptable side-effects will be available in the immediate future. It is an ethical requirement to avoid premature success reports and ill-founded claims that may undermine the legitimacy of biomedical research. Second, scientific and public policy communities should be transparent regarding uncertainties and potential financial and social costs of early diagnosis and treatments in order to foster informed public deliberation and adequate consideration of benefits and concerns.

### Key questions for further debates and research

- Is there a socially accepted wish to know or share knowledge about dementia predisposition? Is there social and cultural diversity about this?
- Given the heterogeneous structure of public healthcare in various countries, what is the overarching professional responsibility in organizing a transnational strategy for care and prevention of dementia?
- What is the responsibility of a nation to promote primary prevention of dementia targeting the healthy population, especially if evidence for effectiveness remains uncertain?

## Ethical issues of research participation

### Rationale

Research into LOAD depends on the participation of large numbers of affected persons at different stages of neuronal decline. Informed consent is considered a necessary precondition. It includes sufficient information, assessment of capacity and understanding, as well as voluntary authorization (Appelbaum, 2007). Since affected persons display different levels of capacity, their involvement poses a range of ethical problems (Kim et al., 2011).

*Pre-symptomatic persons* are usually considered competent. However, they will often not be aware of being in an “at-risk state”. Therefore, their identification as research subjects cannot rely solely on self-recruitment, but requires antecedent measures. As a result, persons might be confronted with information causing psychological distress, posing difficult questions of disclosure, and leading to social stigmatization (Illes et al., 2007). Out of fear, pre-symptomatic persons may also be more vulnerable to therapeutic misconception (Fisher et al., 2012) and might falsely expect a therapeutic benefit of biomarker research. Research subjects should understand the difference between biomarker research and clinical trials. Of course, the value of participating in biomarker research may change over time from primarily altruistic to more personal benefit, as standardization and knowledge improve.

Involving persons with *early stages* of LOAD is important for investigating the standard course of disease and testing the predictive value of biomarkers as well as the efficacy of new therapeutic approaches. However, including these persons poses the problem of assessing decision-making capacity. Depending on type and stage of dementia, this capacity may not be stable but rather fluctuating, and usually declines over time (Karlawish, 2003; Kim et al., 2011). Finally, the assessment of capacity depends on emotional factors, interests, and expectations, and varies between socio-cultural contexts. It requires not only understanding risks and benefits, but also appreciating how research participation will affect one’s own life. People with early LOAD may have deficits that could make it difficult to meet this criterion (Karlawish, 2003).

Involving persons in *advanced stages* of LOAD poses the problems of research with incompetent subjects. Higher safeguards such as minimal-risk conditions and alternative procedures to protect personal integrity and best interest are applied, especially in potentially non-therapeutic research. Notably, there is no international consensus on the definition of minimal-risk conditions and the acceptability of proxy decisions (Kopelman, 2004).

### Recommendations

Furthering research constitutes an honorable objective. However, there are still substantial national differences regarding the development and implementation of ethical guidelines for research with cognitively impaired participants (American College of Physicians, 1989; Council of Europe, 1997; National Bioethics Advisory Commission, 1998). Regardless of the national research setting, the different stages of the potential subjects’ LOAD have to be taken into account. As affected persons’ capacity to consent can change over time, it has to be re-evaluated on a regular basis to decide which informed consent conditions apply.

Studies involving *pre-symptomatic persons* have to observe high standards when dealing with potentially problematic predictive and diagnostic information. Special attention should be paid to questions of disclosing, storing, and passing on this information. Suitable measures should be taken to minimize the risks of psychological distress, familial issues, and social discrimination.

Studies addressing persons with *early stages* of dementia will include many individuals who are clearly capable of providing informed consent and there are existing methods for assessing this and documenting it (Marson et al., 1995). Such methods should be sensitive to cultural differences. Instead of generalizing incapacity of affected persons, supportive tools should be implemented in unclear cases to maintain or enhance decision-making capacity. The informed consent procedure should meet requirements such as use of simple language, repetition of explanation, rehearsals by affected persons and family members, and visual presentations for increasing the understanding of risks and benefits. Furthermore, family members can function as mediators (not as legal proxies) where this is suitable to increase the affected persons’ capacity. Continuing supervision and re-assessment of decision-making capacity appears advisable to detect episodic variation and long-term decline.

Studies involving persons with *advanced forms* of LOAD have to protect the personal integrity and best interest of incompetent subjects. They require careful assessment of risks and benefits to meet minimal risk conditions for non-therapeutic research. Proxy decision-making can involve ethical problems regarding authorization, presumed will, and psychological burden. Alternatively, research advance directives allow the determination of a person’s will with regard to research participation in the case of cognitive incapacity. This necessitates sufficient deliberation and consultations with trained professionals to avoid misunderstandings, as well as a professional and legal framework that ensures adherence. Also, opt-out models should be clarified. Manifestations of assent, continuing self-identity, and “natural will” should be taken into account (Karlawish, 2003; Kim et al., 2011).

LOAD research also offers an opportunity to explore new, innovative models of informed consent. Thus, the realization of a model of “gradual informed consent transfer” could emphasize the development of sustained relationships among subjects/patients, physicians and caregivers, and (an)other significant person(s)/proxy medical decision maker(s). This allows for gradual transfer of consent-giving capacity from the research subject to accompanying others, e.g., during a longitudinal study, and makes it more likely that these “companion proxies” will know and implement the subject’s wishes (Overton et al., 2013).

### Key questions for further debates and research

- How can we detect and overcome potential loopholes such as therapeutic misunderstanding in existing informed consent practices given a high risk of fear and psychological distress in connection with a diagnosis of (pre-)dementia?
- Given the complexity of information on biomarkers and dementia, how can subjects’ understanding of information regarding research, diagnostics, and treatment be improved?
- How can we overcome a narrow focus on individual autonomy and consider ethically and legally the role of the family and the social practice of knowledge-sharing in pre-dementia research?

## Individual and social consequences

### Rationale

Predictive and diagnostic information on LOAD can open possibilities of adapting life plans and making provisions. It may also provide psychological relief and social exoneration. On the other hand, it can pose difficult ethical problems:

On the *individual level*, this information can lead to psychological distress and irreversible decisions, even up to pre-emptive suicide, particularly when no efficacious therapies are available. It can affect persons’ perceptions of themselves and their families, leading to negative self-images. The REVEAL-study on ApoE-genetic testing actually suggests that impacts might be less severe (Green et al., 2009). However, some studies indicate that lay people consider genetic information as less decisive than, e.g., family history (Chilibeck et al., 2011). Others point at considerable cultural or even national differences regarding peoples’ interests to pursue genetic risk assessment for AD (Alzheimer Europe, 2011). Similar studies for neuro-biomarkers are needed to assess whether genetic and neuroscientific information differ from a lay person’s perspective. If the predictive value of biomarkers is better than ApoE-genetic tests, their individual and social implications might differ.

In the *family context*, prognostic information can cause serious disruptions, especially when inheritable risk-factors may be involved. This poses the question of who else might be concerned, directly or indirectly. Thus, the affected person is confronted with disclosure dilemmas between privacy, protection, and family responsibilities (Rehmann-Sutter and Müller, 2009).

On a *societal level*, an increasing gap between prognosis/diagnosis and treatment can be accompanied by stigmatization. This can lead to the classification of pre-symptomatic risk-bearers, overburden them with responsibilities, and result in their medicalization. It can also promote their discrimination (e.g., with regard to legal status, job positions, or access to health insurance) and commercial exploitation, e.g., through dubious direct-to-consumer testing (Garand et al., 2009).

### Recommendations

In dealing with predictive information, highest professional and legal standards should be observed to protect subjects from psychological distress, moral dilemmas, and social stigmatization. This information should be used to empower affected persons and optimize their treatment and care. Attempts to exclude them from social participation, healthcare, or other resources must be inhibited. Professional guidelines should be based on more empirical, cross-cultural studies on how lay people actually deal with LOAD risk prediction.

In a future scenario of clinical implementation of research results, the subjects would have to be informed in advance about all relevant possible research outcomes and their potential implications. Their right not to know would have to be respected (e.g., by offering an option to waive claims to be informed). However, this presents practical and ethical challenges for clinical drug trials. Subjects would need to agree before entering the study that they would find out their biomarker status as it may be unethical to expose participants who do not express the targeted biomarkers to drugs. If information were disclosed, professional counseling would have to be provided. This should include a critical revision of the information’s reliability, significance, and implications for family members in order to prevent rash and inconsiderate reactions such as pre-emptive suicides. Furthermore, practical coping strategies and concrete problem solutions have to be delineated. To avoid stigmatization and discrimination, restrictive standards of confidentiality and data protection for any biomarker test including imaging techniques would be needed. Professional practice should take into account cultural diversity in dealing with such information. Given the globally diverse population and differences in legal systems, attempts are needed to foster a dialog between science and law. In countries with low levels of informed consent, counseling, or data protection, the scientific community should promote high standards for clinical application. Finally, scientists and policy makers investing in profit seeking biomedical industries gaining from this research should avoid conflicts of interest. Legal regulations are needed to preclude an unhampered market of direct-to-consumer testing without sufficient evidence-base or adequate supervision and counseling.

### Key questions for further debates and research

- How can ethical and clinical guidelines be respectful and sensitive to diverse ethnic populations having often very different understandings of family hierarchy, gender, and age norms?
- How can international consensus about data protection and anti-stigmatization strategies be ensured (e.g., by international associations)?
- How do we gain a minimum international ethics standard for research to ensure ethically acceptable transnational cooperation and to avoid “ethics dumping” (i.e., outsourcing of research into countries with lowest ethics standards or missing control)?

## Exploring diversity and promoting public dialog

### Rationale

While the neuroscience community is increasingly globalized, cross-national comparison reveals significant differences in images, practices, and policies regarding LOAD. Some national policies emphasize individual suffering and public health burden, others dignity, quality of life of affected persons, and societal solidarity. Correspondingly, the focus of public discourses, interest group strategies, and governmental policies can be on supporting biomarker research or rather on providing adequate care for patients in advanced stages. Diversity presents a practical and economic challenge in dealing with research subjects from different cultural backgrounds. At the same time, it constitutes a resource for problem solving of complex human issues (Illes et al., 2007).

### Recommendations

Since biomarker research still is in an early phase, it is a critical point in time to envision the spectrum of implications for dealing with LOAD. An intensified dialog between neuroscience, social sciences, and humanities will increase the understanding of the diversity and intersectionality (cross-influences of cultural categories such as gender, class, religion) of cultural attitudes towards LOAD and healthcare. Researchers and clinicians should be careful not to impute their own attitudes without critical consideration. Deliberative participatory processes in setting research and healthcare policy priorities should be strengthened to include the different perspectives and to benefit from the abundance of approaches.

The involvement of those affected is a constitutive feature of modern science governance that recognizes the right to self-determination but also aims at empowering patients and their families to take a more active role in shaping science (Schicktanz et al., 2012). Patient associations are important mediators, but a balanced plurality of advocacy groups should be included in order to avoid a one-sided perspective (e.g., by over-representing or neglecting either patients or their care-givers). Overall, framing dementia only as a threat to social and healthcare systems should be avoided, since this increases the stigmatization of patients and their families. Further research into causes, prevention, and treatment of LOAD, as well as adequate and respectful care for those affected, are necessary. Both constitute morally justified and valuable concerns that should not be pitted against each other. More national and international scientific societies should function as mediators and dialog platforms to foster an interdisciplinary and socially engaged discourse on the aims of LOAD research.

### Key questions for further debates and research

- How can we organize public deliberation that adequately involves the perspectives of those already affected by LOAD?
- How can we foster a dialog between various experts from bioscience, clinical care, social gerontology, ethics, and public health research in each country?
- How can the dominance of one-sided negative images and metaphors of dementia (e.g., “threat”, “epidemic”, “loss of self”, “public burden”, “living death”) be overcome?

## Author statement

All authors made substantial contributions to the conception or design of the work, drafted the work or revised it critically for important intellectual content, and gave their final approval of the version to be published. They agree to be accountable for all aspects of the work in ensuring that questions related to the accuracy or integrity of any part of the work are appropriately investigated and resolved.

## Conflict of interest statement

The authors declare that the research was conducted in the absence of any commercial or financial relationships that could be construed as a potential conflict of interest.
